# Musculoskeletal ultrasonography in rheumatic diseases

**DOI:** 10.55730/1300-0144.5723

**Published:** 2023-07-20

**Authors:** Erhan ÇAPKIN

**Affiliations:** Department of Physical Medicine and Rehabilitation, Faculty of Medicine, Karadeniz Technical University, Farabi Hospital, Trabzon, Turkiye

**Keywords:** Musculoskeletal ultrasonography, rheumatoid arthritis, spondyloarthropathies, crystal arthritis

## Abstract

Ultrasonography is an imaging technique based on sound waves used for the evaluation of soft tissues. Sound waves have been used for a long time in nonmedical fields, including military defense systems, radar systems, and detection of icebergs. Technological advances resulted in new techniques becoming available for medical imaging, including ultrasonography, magnetic resonance imaging, and computed tomography. Nowadays, the use of imaging has become a gold standard protocol in the diagnosis of many diseases, and recently developed diagnosis and therapy options provide more efficient treatment of rheumatic diseases. Thus, it has become possible to prevent structural damage and disability in patients with rheumatic disease. Musculoskeletal ultrasonography is becoming a preferred imaging technique for rheumatic diseases, as it has many advantages. Among its advantages are being inexpensive, being radiation-free, having a dynamic image capacity, helping to detect disease activity, and helping with early detection and diagnosis of structural damage. This review summarizes the use of ultrasonography in rheumatic diseases.

## 1. Introduction

Ultrasonography (US) is based on the creation of sound waves at a frequency that the human ear cannot hear that are sent to the body through probes and reflected from the tissues as images on a screen through an operating system. This principle is called the piezoelectric principle. The use of sound waves in imaging goes back a long time. It has been used in nonmedical fields, including defense systems, radars, and for the detection of icebergs, and in the 19th century, it started to be used in medicine and health services [1]. A study was published for the first time in the 1940s about the use of US for the detection of breast nodules. Later, US started to be used for diagnosing ovarian cysts, and many clinicians started to publish papers about the use of US in their own branch of medicine [2]. The first published article on its use in musculoskeletal diseases was published in 1972, where it was used for the differential diagnosis of a Baker’s cyst with thrombophlebitis [3]. The sound waves reflected from tissues in US are transferred to an operating system via a probe. The image is formed in a range of black and white colors according to the reflective properties of the textures. The image formed in ultrasonography is described by the concept of echogenicity. Liquids are seen as black (anechoic) because they pass all sound waves, while bone tissue is seen as white (hyperechoic) because it reflects all the sound waves that it is exposed to (Table 1).

There are various modalities in US evaluation. Gray scale is the most used mode. Also known as B mode, the image is in the range of black and white. Color Doppler showing blood flow and M mode are mostly used for the cardiovascular system. Power Doppler (PD) mode is used in rheumatology to show the smaller microvascular bed, especially the synovial blood supply. PD is particularly important in demonstrating disease activity and determining prognosis.

Probes with many different features are used in the evaluation of US. Linear probes are generally sufficient to evaluate the musculoskeletal system. Convex probes can be preferred to evaluate deep tissues such as hip and sacroiliac joints. Although the frequency range of the probe varies according to the tissue to be imaged, high frequency probes should be used for superficial tissues and low frequency probes for deep tissues [1]. Increasingly, US has been used by clinicians across the many stages of rheumatic disease treatment, such as for diagnosis, follow-up, and interventional procedures, especially for inflammatory diseases. Various recommendations regarding the use of US in rheumatic diseases have been presented in many international guidelines.

### 1.1 Detection of pathologies by ultrasound

The study group named Outcome Measures in Rheumatoid Arthritis Clinical Trials (OMERACT) defines the outcome measurement methods used in clinical studies in rheumatology. In 2005, a definition proposal was made for the pathologies seen using US for inflammatory arthritis. This recommended is currently used in patient descriptions [4]. According to the recommendations of this study group, the pathologies seen in inflammatory diseases are defined as follows:

#### Synovial fluid

**Figure 1:**
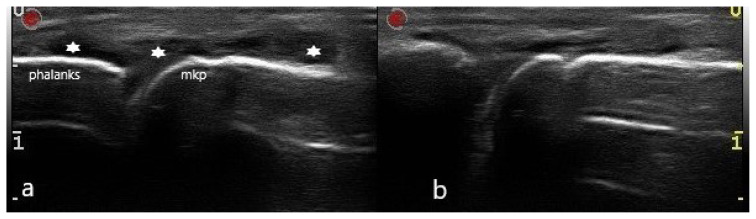
An anechoic effusion in the second MCP joint in an RA patient (a), and fluid disappearing when pressure is applied to the skin with a US probe (b). The stars indicate synovial fluid, “mkp” is the metacarpophalangeal joint, and “phalanks” is phalanx.

Abnormal hypoechoic or an anechoic (relative to subdermal fat, but sometimes isoechoic or hyperechoic) intraarticular material that is displaceable and compressible, but does not exhibit a Doppler signal (Figures 1a and 1b)

#### Synovial hypertrophy

**Figure 2:**
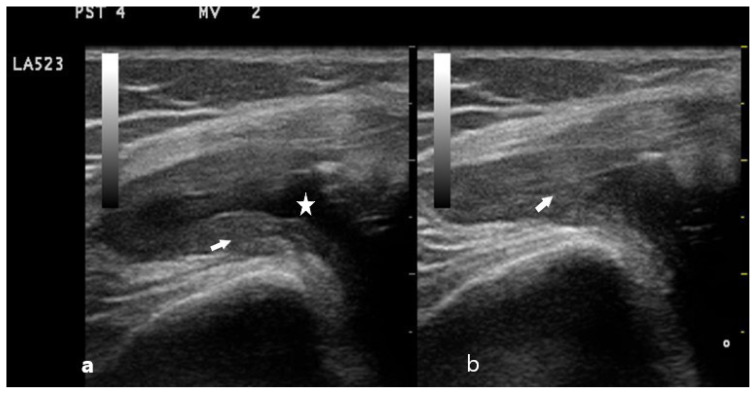
The joint fluid has disappeared with compression (a), but the synovial tissue is not displaced and has a hypoechoic appearance (b). The stars indicate synovial fluid, and the arrow is a synovial hypertrophy.

Abnormal hypoechoic (relative to subdermal fat, but sometimes isoechoic or hyperechoic) intraarticular tissue that is nondisplaceable, poorly compressible, and may exhibit a Doppler signal in relation to disease activity (Figures 2a and 2b).

#### Enthesitis

**Figure 3:**
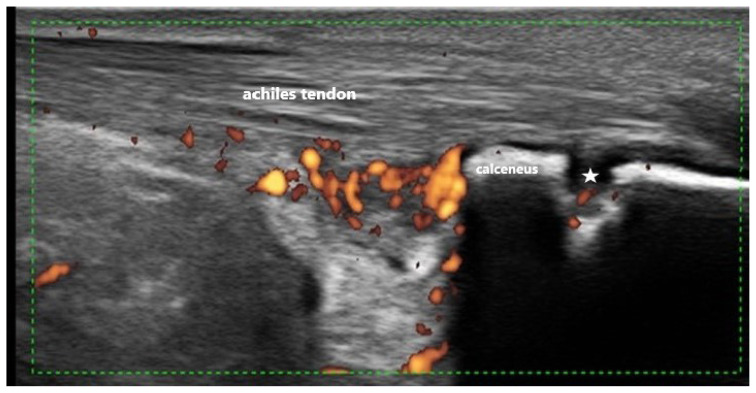
Thickening of the tendon and a hypoechoic appearance at the Achilles attachment site, with calcaneal erosion and increased Doppler activity involving the retrocalcaneal area. The star indicates the calcaneal erosion.

Bone changes such as abnormal hypoechoic appearance (loss of normal fibrillar structure), thickening (sometimes may include hyperechoic foci compatible with calcification), cortical bone loss, and new bone formation in the bone adhesion area of the tendon or ligament. It can give a Doppler signal (Figure 3).

#### Tenosynovitis

**Figure 4:**
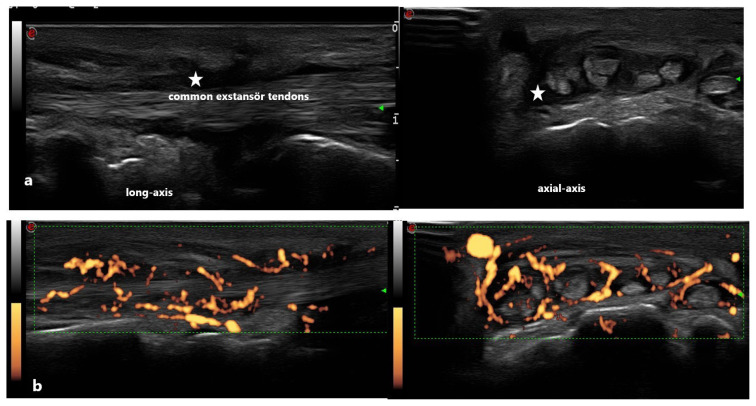
Tenosynovitis image of an RA patient at wrist level shows active (grade 3) tenosynovitis with both axial and longitudinal gray (a) and Doppler scales (b). Stars indicate synovial fluid.

In both planes, hypoechoic or anechoic thickening of the tendon with or without anechoic synovial fluid around the tendon. It is associated with disease activity and may exhibit Doppler activity (Figures 4a and 4b).

#### Erosion

**Figure 5:**
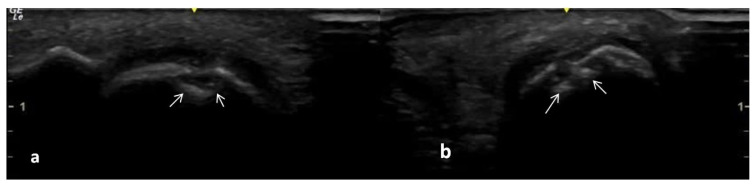
Erosion in the 5th MTP foot joint of an early RA patient. There is cortical continuity deterioration in two planes (a: longitudinal; b: transversal scans). The arrows indicate erosion.

The loss of cortical continuity on the bone surface. This cortical discontinuity should be shown in both axial and longitudinal imaging (Figures 5a and 5b).

A semiquantitative scoring system is often used to determine disease activity, both in gray scale and PD scale. Synovitis and tenosynovitis are generally classified separately. In this system, they are defined between 0 and 3 points as normal, mild, moderate, and severe. In a very recent classification for rheumatoid arthritis, gray scale and Doppler scale have been combined (Table 2) [5].

## 2. Ultrasonography for rheumatoid arthritis

**Figure 6:**
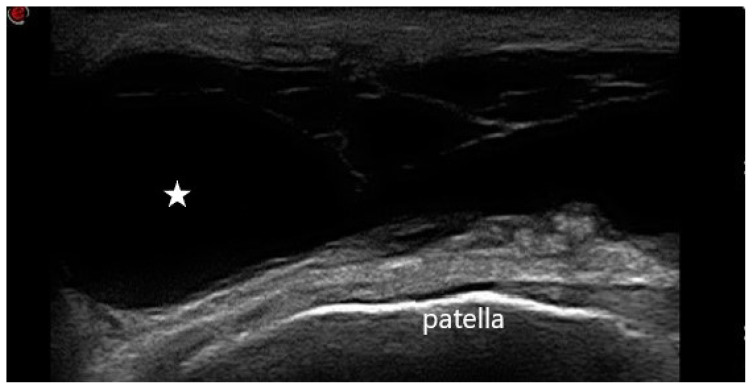
US image of a 42-year-old patient with complaints of pain and swelling in the knee and diagnosed with prepatellar bursitis. The hypoechoic line is below the patella, and the bursa containing anechoic fluid material is above the patella. The star indicates synovial fluid.

Rheumatoid arthritis (RA) is the most common inflammatory rheumatic disease. This disease, which starts in the synovium, affects all the structures of the joint and causes structural damage and disability. Early recognition and treatment of the disease reduces the risk of structural damage. Effusion, synovial hypertrophy, tenosynovitis, and erosions seen in RA patients can be detected at an early disease stage with US. The first regions usually involved in RA are the wrists, metacarpophalangeal (MCP), and metatarsophalangeal (MTP) joints [6]. The main criterion for applying the classification criteria made according to the 2010 European Rheumatology Association (EULAR) and American Rheumatology Association (ACR) criteria is the presence of synovitis in a joint [7]. Here, swelling is an important criterion. The 2016 EULAR early arthritis guideline recommends that if arthritis is suspected in a joint, it should be confirmed by ultrasonography [8]. Studies have shown that US is superior to clinical examination in detecting fluid in the joint. Especially in individuals presenting with the complaint of joint pain, US makes significant contributions to the question of whether the pathology is caused by extraarticular structures (Figure 6). Synovitis is the most basic finding of RA. Early synovitis is defined as the period when synovial inflammation begins but clinical signs do not appear. As a result of proliferative synovitis, the synovial membrane thickens to become a hypertrophic and hypervascular tissue, and structural damage begins to occur with many proinflammatory cytokines released into the area. At this early stage of the disease, it is impossible to evaluate these changes in soft tissues with X-rays, so US can provide valuable information. Early RA studies on synovitis found that with US, sensitivity increased to 78% and specificity to 79%, while clinical examination had sensitivity of 58% and specificity of 78%; moreover, the US specificity increased even further if the synovitis was considered US grade 2 or higher for patients with US grade 1 synovitis scores [9]. In this respect, US has a very important place in detecting synovitis in the early stage of the disease. In a study conducted in another cohort Investigators of the French Early Arthritis Cohort (ESPOIR), US sensitivity was 74% and specificity was 90% for patients with a synovitis score of grade 2 or higher [10]. The results of many studies indicate that US is a superior imaging method compared to clinical examination and X-ray at the early stage of the disease. In addition, information about the severity of synovitis can be obtained with PD activity [11], and the findings obtained with US correlate to magnetic resonance imaging (MRI) in comparative studies[12].

Disease activity is very important for the development of structural damage. The number of sensitive or swollen joints and acute phase indicators can be used to predict disease development. US helps to detect the activity of the disease, particularly as PD signals indicating increased blood supply and its severity correlate with the activation of the disease. In many studies, activation detected by US correlated with disease activity [13–15]. Furthermore, subclinical inflammation in patients with RA can be demonstrated with US. The risk of relapse has been found to be higher in those with positive PD signals, especially in studies conducted in patients in clinical remission [16]. The main characteristic of imaging among RA patients is the development of erosion. US can show erosions at an early stage, before being visible on an X-ray, and Backhaus et al. have shown in a clinical study that US detects erosions better than X-rays [17]. Subsequently, US was shown to be superior in detecting erosion, especially in the early disease stage, in many studies [18,19]. Many studies have been conducted on the use of US for disease monitoring and evaluation of treatment response. Filippucci et al. evaluated patients using US before and after intraarticular steroid injection, and they found a decrease in Doppler signal as well as in clinical response [20]. In a similar study, Hau et al. ultrasonographically demonstrated the reduction in synovial vascularization after antiTNF treatment [21]. Similar results have been shown in different studies of biological agents [22–24], so it seems that US can be used effectively to evaluate the treatment response of the disease. In a disease with progressive characteristics, such as RA, markers related to its progression are very important. Disease activity, the number of swollen joints, auto antibody positivity, and the presence of erosion are the most important prognostic markers [25]. Many studies have been conducted on the prognosis of the disease with US. Taylor et al., in a study conducted using US for 24 early RA patients, showed that the patients with a significant Doppler signal in the early stage of the disease had more radiographic damage after 2 years of follow-up. They concluded that the existing Doppler signal in patients may have a predictive importance for the development of structural damage [26]. In a similar study, Naredo et al. found a positive correlation between US scores, disease activity scores, and radiographic progression in early RA. In another study by the same team with more patients, they showed that the PD signal was blinded by activity and radiological progression [16]. Brown et al., in a study of 102 RA patients with subclinical disease activity, showed that patients with MCP join synovial hypertrophy and Doppler signal progressed radiographically [27]. Although the use of US in RA patients has many advantages, its use to evaluate which joints have polyarticular involvement in a disease is controversial. However, different joint numbers have been defined in many joint scoring systems [28–31]. Scheel et al. noted 3 joints (the 2nd and 4th MCP joints and the palmar surfaces of the proximal interphalangeal (PIF) joints) [32], and Naredo et al. suggested 12 joints (the 2nd and 3rd bilateral MCP, the wrist, the 2nd and 3rd PIF, and the knee) [33]. Backhaus et al. noted that evaluation of the wrist, the 2nd and 3rd MCP and PIF joints, and the 2nd and 5th MTP joints would be sufficient for the hand and foot, and recommended examining the intensity of the gray scale synovitis, tenosynovitis, and Doppler signal [34]. They used the German US 7 score and proposed this scoring system for use in daily practice and studies [35]. Considering the scoring system, their recommendation for daily practice was to first evaluate the joints which the patient complained about, and then do a bilateral examination of the most frequently affected joints: the wrist, the 2nd and 5th MCP joints in the hand, and the 2nd and 5th MTP joints in the foot, both in gray scale and Doppler and in terms of signal.

**Figure 7:**
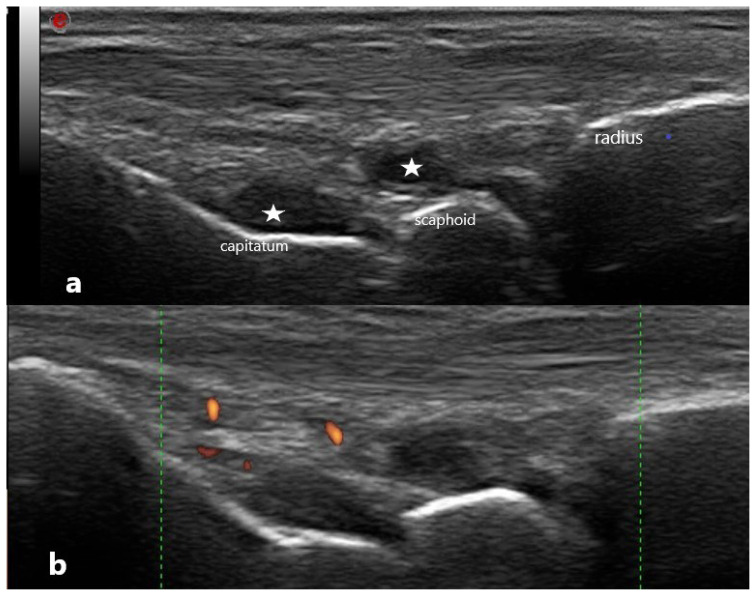
US image of the wrist of a 36-year-old patient with a 5-year diagnosis of RA, with no joint sensitivity or swelling, and using methotrexate 20 mg/week and hydroxychloroquine 200 mg/day. Sedimentation rate is 2 mm/s, CRP is 0.1 mg/dL, DAS is 28, ESR is 0.49, SDAI is 2, and CDAI is 2. Gray scale (a) shows fluid in the joint space, and there is a weak Doppler signal on the wrist (b). This patient is in remission clinically but not sonographically. The stars indicate synovial fluid.

US is used as a guide for local injections for many musculoskeletal diseases, and it has been found that US-guided injections are more effective and the risk of side effects is lower with this approach [36,37]. Similar results have been shown in injection studies for RA [38–41]. It is stated in the EULAR RA imaging recommendations that can be used in the evaluation of RA remission. In a metaanalysis of 1639 patients from 19 studies on this subject, most (1369) were in clinical remission. While 80% of these patients had US findings in the gray scale, 44% of them had positive synovitis in both the gray scale and the PD scale, which raises the question of whether clinical remission in these studies is true remission. Although the debate on this issue continues, relapse rates are higher, especially in patients with positive PD signals. In this respect, it may be an option to evaluate patients with US when considering drug reduction or elimination, but more clinical studies are needed on this subject (Figures 7a and 7b) [42]. In two studies on the use of US in treatment management, the role of US was taken into consideration while deciding on the choice of treatment [,]. One hundred twenty-two patients were randomized to an ultrasound tight control strategy targeting clinical and imaging remission, and 116 patients were randomized to a conventional tight control strategy targeting clinical remission. Patients in both arms were treated according to the same disease modifying antirheumatic drug escalation strategy, with 13 visits over 2 years. Twenty-six (22%) of the 118 analyzed patients in the ultrasound tight control arm and 21 (19%) of the 112 analyzed patients in the clinical tight control arm reached the primary endpoint (mean difference 3.3%, 95% confidence interval −7.1% to 13.7%). While there was no significant difference in activity score in the US arm compared to the clinical arm, the US arm showed less progression in radiological scores. More medication was used in the US arm. Although the role of US in making treatment decisions is controversial, it has been emphasized that studies with larger populations are needed[43]. To summarize, as emphasized in the EULAR imaging guideline, US is an increasingly important imaging method for the management of many stages of RA patients, including diagnosis and follow-up treatment selection [44–45].

## 3. Ultrasonography for spondyloarthropathies

There has been a significant change in the terminology of spondyloarthropathies (SpA) in recent years, such that this group of diseases has been classified into two categories. One is axial spondyloarthritis (ankylosing spondylitis and nonradiographic axial SpA), which has more involvement of the axial spine, and the other is peripheral SpA (psoriatic arthritis, reactive arthritis, undifferentiated arthritis, and inflammatory bowel disease-related arthritis), which has more prominent peripheral involvement. Many methods are used to monitor these diseases. Although MRI is the gold standard method for axial disease, US is a very important method in the evaluation of arthritis, enthesitis, tenosynovitis, and dactylitis in peripheral involvement [464]. The EULAR SpA imaging guideline recommends using US for the diagnosis, follow-up, and evaluation of structural damage in peripheral SpA [47]. Evaluation of synovitis, synovial hypertrophy, effusion, and Doppler activity in peripheral SpA is done in a similar way to RA. Tendon involvement and the tendon–bone junction are considered very important target organs for SpA, and US is the gold standard method for the evaluation of these structures. When there is disruption in the tendon fibrillar structure and tendon thickening, hypoechoic areas and increased Doppler activity are observed. Dactylitis is an important clinical finding, especially in PsA patients. In addition to joint involvement, soft tissue edema and the thickening of tendons can be seeing using US [48]. Enthesitis is a characteristic finding of SpA patients. Pain and tenderness at the bone attachment site of tendons and ligaments are typical findings mostly seen in the Achilles region. Clinically, palpation sensitivity in this region is the most basic examination finding. US evaluation of this region is very important for active SpA patients. According to the International Society of Ankylosing Spondylitis (ASAS) and the Psoriasis and Psoriatic Arthritis Working Group (GRAPPA), the presence of enthesitis is considered both a diagnostic and an activation indicator. The first study with US, by Lehtinen et al. in 1994, showed changes seen in this region by US on a gray scale. Since then, many studies have been conducted on the use of gray scale and Doppler scale together [49]. In the early stage of the disease, in differential diagnosis (especially from fibromyalgia), tendon echogenicity deterioration and hypoechoic areas increase in Doppler activity, and erosions in the bony cortex are observed in US [50]. Although many studies have shown a correlation between disease activity, clinical enthesitis scores, and US images, some articles emphasize that there are conflicting results, and often there is no consensus on the assessment of the patient [51–53]. The use of various scoring systems for the assessment of the enthetical region has been proposed [54]. As a result, the use of US in the diagnosis of SpA, especially in the diagnosis of peripheral involvement, is considered to greatly contribute to the clinician. The use of US is recommended in the EULAR imaging recommendations.

## 4. Ultrasonography for crystal arthropathies

**Figure 8:**
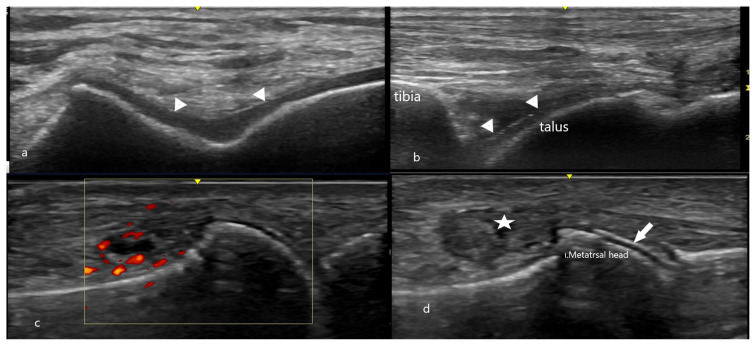
MSU crystals deposited on the cartilage surface: distal femur cartilage (a), talus cartilage (b), active gout arthritis, positive Doppler signal (c), and double contours seen in the 1st MTP joint in a gout patient (d). Arrowheads indicate double contours, and asterisks indicate a snowstorm.

**Figure 9:**
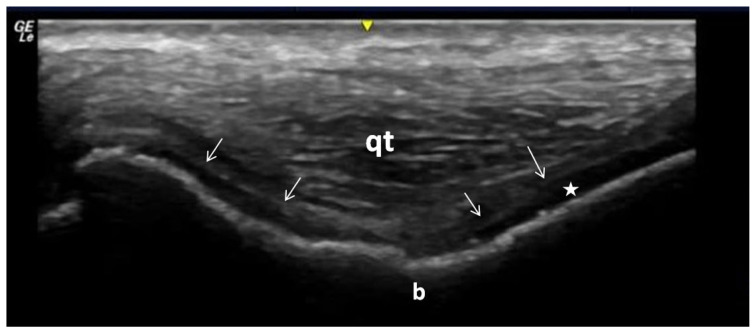
Calcium pyrophosphate dihydrate crystals accumulated in the hyaline and fibrocartilage in the knee joint. Unlike in gout, these crystals accumulate mostly inside the cartilage. The star indicates cartilage, the arrows show calcium pyrophosphate dihydrate crystals, qt: Qudriceps tendon and b is the distal femur bone.

US is frequently used in the diagnosis of crystal-associated arthritis. When crystal arthritis is mentioned, the first disease that comes to mind is gout, in which monosodium urate (MSU) crystals accumulate. It is one of the most common inflammatory diseases. In its pathogenesis, the disease develops because of the accumulation of urate crystals in the joints and surrounding structures, triggering the inflammatory process. The definitive diagnosis is to see these accumulated crystals microscopically, but this may not be possible for every patient; crystals may not be visible under a polarized microscope in 25% of patients [55]. In recent years, new methods of imaging have been used to facilitate the diagnosis of this disease. Dual energy computed tomography (CT) and US are the leading methods, as these crystals are not visible on X-rays. With US, these crystals are seen as hyperechoic and shiny. Symptoms of synovitis that develop in the early stage of the disease are seen in the joint space as increased joint fluid, synovial hypertrophy, and Doppler activity [55]. Urate crystals usually accumulate on the cartilage surface, and the US in this case is typically defined as a double contour image (Figures 8a–8d) [56]. The OMERACT group has defined 4 main US findings of gout, namely double contours, tophi, aggregates, and erosion. The double contour is the image formed by the crystals deposited on the joint surface. Unlike calcium pyrophosphate, urate accumulates on the joint surface. Aggregates are crystal deposits that do not give acoustic ghosting and are deposited in the joint space or in the soft tissue. Tophi accumulates in the joint or soft tissue, and acoustic shadowing is usually not observed. Erosion in gout is perceived to be an elementary lesion. The applied ultrasonographic definition of bone erosions in gout is the same as the definition of bone erosions in rheumatoid arthritis. However, bone erosions in gout patients are, in contrast to those in other arthropathies, commonly found extraarticularly. Therefore, the distribution of bone erosions in gout patients, rather than the appearance of a single erosion, makes the erosion characteristic for gout [57]. In the new gout classification criteria, US findings are given a scoring point value [58,59]. In studies conducted on using US findings for the diagnosis of gout, the specificity of the double contour image was found to be quite high (98%), which studies on its sensitivity reported results ranging from of 22% to 91% [60,61]. Erosions seen on X-rays in the late stages of the disease can be detected earlier with US, which has been shown to be more sensitive in comparative studies with conventional radiography [62]. Studies on MRI and CT have shown that these two methods are superior to US in detecting erosion [63]. However, US can be used in the follow-up of the disease, especially for following double contours and tophi. Small studies have shown regression in US findings after urate-lowering therapy [64]. Clinical response to US after colchicine treatment was found to correlate with clinical response in the joint, as well as decreased US findings [65]. Another crystal storage disease, calcium pyrophosphate dihydrate disease, can be seen by US from the deposited crystals (Figure 9). In the early stages, deposits that cannot be seen by X-ray can be identified by US. Unlike in gout, the accumulated crystals are seen in the cartilage and meniscal structures [66,67]. Studies comparing US with X-ray showed that US is superior, especially in the early period. Interestingly, although there is calcium in the accumulated crystals, unlike other calcific deposits, postacoustic shading is not seen in these crystals [68,69]. There is more deposition in the fibrocartilage structures than in the hyaline cartilage [70].

## 5. Ultrasonography for polymyalgia rheumatica

Polymyalgia rheumatica is a common inflammatory rheumatic disease characterized by a high sedimentation rate, high C-reactive protein (CRP) levels, pain, and stiffness in the muscles of the shoulder and hip girdle. Diagnostic difficulties may be present in patients with an unclear acute phase response. US is useful in diagnostics for these patients, and disease activation correlates with US findings. Using US, fluid can be seen in the biceps tendon sheath, glenohumeral joint, and hip joints. Tenosynovitis and Doppler activity may be increased, and these are indicators of disease activation [71]. Alongside clinical findings, US findings are included in the classification of new diagnostic criteria [72].

## 6. Ultrasonography for degenerative diseases

**Figure 11:**
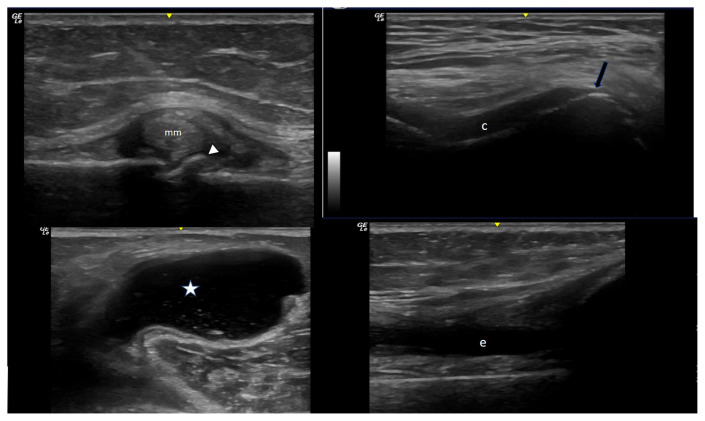
Sonographic findings in knees with OA include osteophytes (white arrowheads), suprapatellar effusion (e), protrusion of the medial meniscus (mm) with displacement of the medial collateral ligament, a Baker’s cyst (star), and decreased cartilage (c) thickness (arrow).

X-ray still maintains its usefulness for degenerative joint disease, but thinning of cartilage, increase in joint fluid, and osteophyte formations can be seen in US (Figure 11). Calcium crystals may be seen accumulating in osteoarthritis (OA) patients, and US guidance is often used for local injections [73]. Although EULAR recommends the use of US in OA imaging, especially in the early stage of the disease, during synovitis attacks, for showing soft tissue changes, and for injection guidance, X-rays are still useful for OA patients. It has been stated that advanced imaging methods can be used in atypical cases, such as rapidly progressive patients, and in the above-mentioned conditions [74].

## 7. Ultrasonography for other rheumatic diseases

In addition to its use in rheumatic diseases that are frequently seen in daily practice, US is used today in rheumatic diseases that are less common. In these cases, it helps in diagnosis, especially in large vessel vasculitis. Temporal artery US helps diagnosis of giant cell arteritis. Homogeneous thickening of the vascular wall and hypoechoic areas as well as decreased blood flow are findings that can be seen with US 75,76]. In Sjögren’s syndrome, impaired echogenicity in the parotid and submandibular glands and the presence of hypoechoic foci help the diagnosis. Adding sonographic findings to the clinical diagnostic criteria increases the diagnostic probability [77]. In systemic sclerosis, skin evaluation can be performed with high-frequency probes [78].

## 8. New US modalities

In addition to the gray scale and PD methods commonly used today, there are new developments in US, with 3D imaging, elastography, and hybrid systems being the main ones. 3D imaging is a new US modality that can be used especially for detecting erosions and quantitative measurement of Doppler activity [79]. Elastography may provide additional contributions, especially in the evaluation of tendon pathologies, at an earlier stage. MRI or CT and US can be applied simultaneously. These methods are not currently in clinical practice but are new promising methods [80].

## 9. Advantages and disadvantages of US

**Figure 12:**
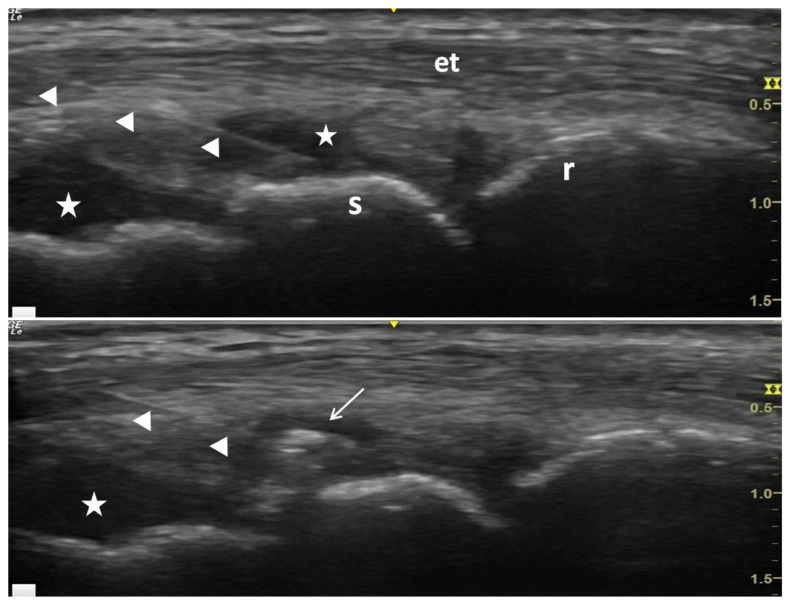
Wrist injection in RA patients (a). The arrowheads show the needle, r is the radius, s is the scaphoid, et is extensor tendon, the stars indicate synovial fluid, and the arrow in (b) shows the postinjection steroid crystal deposits.

US is the most practical and fastest method for imaging the musculoskeletal system. It can be easily applied in the clinic, multiple joints can be evaluated in real time, and it allows for dynamic examination of tissues. All structures located on the bone can be evaluated, depending on the capacity of the device and the characteristics of the equipment used. It can show needles and target structures in real time in interventional procedures. It enables the clinician to perform an effective interventional procedure without damaging the surrounding tissues and causing radiation exposure (Figures 12a and 12b). It provides the opportunity to make simultaneous comparisons with normal tissues, and it contributes to the increased effectiveness of applied treatments as it gives the possibility to recognize many pathologies at an early stage. Alongside these many advantages, the biggest limitation is that it is more user-dependent than other imaging methods, so the experience and training of the practitioner is very important. Moreover, its inability to obtain multiple planed images or show the inside of the bone, the presence of anatomically inaccessible areas of sound waves, and the time needed for clinical examination are further limitations [81].

## Figures and Tables

**Figure 10:**
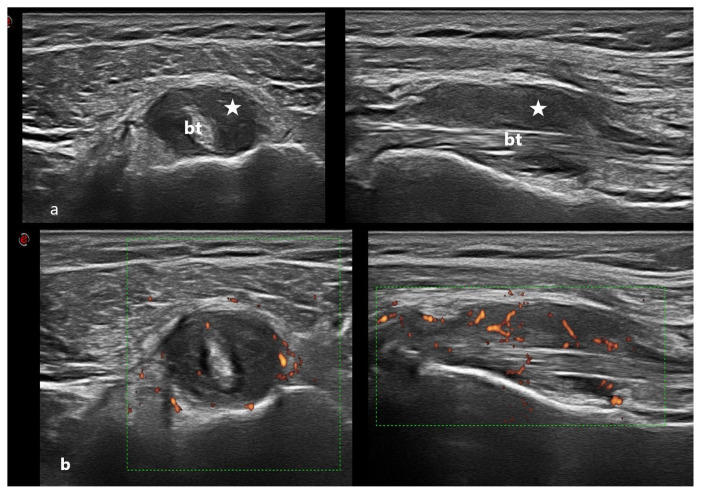
Gray scale (a) and Doppler scale (b) US performed on a shoulder with PMR diagnosis, the patient has edema and an anechoic effusion area around the shoulder with a thickened biceps tendon in both planes (axial and longitudinal). Stars indicate synovial hypertrophy, and bt shows the biceps tendon.

**Table 1:** Ultrasonography image color scale.

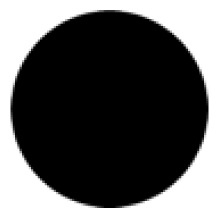	anechoic	fluid, articular cartilage
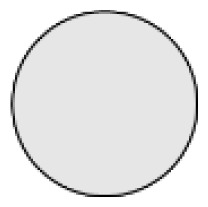	hypoechoic	synovial tissue, muscle fibril*, nerve*
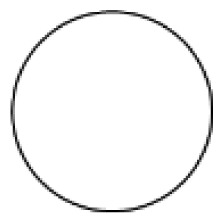	hyperechoic	bone, tendon*, fat, calcification, fibrocartilage*

* These can vary depending on the reflection of sound waves and surrounding tissues.

**Table 2:** Ultrasonographic semiquantitative scoring with combined gray scale and PD scale evaluation.

Grade	Description	Gray scale	Power Doppler (synovium)
0	Normal	0	0
1	Mild	1	≤1
2	Moderate	2	≤2
3	Severe	3	≤3
